# Role of immunoglobulin in neuronal apoptosis in a neonatal rat model of hypoxic ischemic brain injury

**DOI:** 10.3892/etm.2014.1470

**Published:** 2014-01-02

**Authors:** SALIH KALAY, OSMAN ÖZTEKIN, GÖNÜL TEZEL, HAKAN ALDEMIR, EMEL SAHIN, SADI KÖKSOY, MUSTAFA AKÇAKUŞ, NIHAL OYGUR

**Affiliations:** 1 Department of Pediatrics, Division of Neonatology, Akdeniz University Medical School, Antalya 07070, Turkey; 2 Pediatric Surgery, Anadolu Hospital, Antalya, Turkey; 3 Organ Transplantation Research Laboratory, Akdeniz University Medical School, Antalya 07070, Turkey; 4 Department of Medical Microbiology, Akdeniz University Medical School, Antalya 07070, Turkey

**Keywords:** hypoxic ischemic encephalopathy, cytokine, apoptosis, intravenous immunoglobulin

## Abstract

The objective of the present study was to evaluate the neuroprotective effects of immunoglobulin (Ig) in a neonatal hypoxic ischemic (HI) rat model. Seven-day-old rat pups were randomly assigned to control, hypoxia and hypoxia + Ig groups. The rats in the hypoxia +Ig group were intraperitoneally administered 1 g/kg Ig once, immediately after hypoxia. Saline was administered to the rats in the hypoxia group at the same time point. Eight rats from each of the Ig + hypoxia and hypoxia groups were sacrificed by decapitation 4 and 24 h following the administration of Ig or saline. The rats of the control group were sacrificed at the 4 h time-point. Caspase-3 activity, as well as IL-1β, IL-6 and TNF-α mRNA expression levels, were studied in the left ischemic hemispheres. Induction of cerebral ischemia increased the TNF-α, IL-6 and IL-1β mRNA expression levels significantly at 4 and 24 h in the left ischemic hemispheres in the hypoxia group compared with those in the control group. The systemic administration of Ig following HI encephalopathy significantly reduced the TNF-α, IL-6 and IL-1β mRNA expression levels in the ischemic tissue in the Ig + hypoxia group compared with those in the hypoxia group. In the hypoxia group, caspase-3 activity in the left half of the brain was found to be significantly increased compared with that in the control group. Caspase-3 activity in the Ig + hypoxia group was significantly lower than that in the hypoxia group. The observations of the present study indicate that Ig administration may be an efficient treatment approach for reducing cerebral apoptosis associated with hypoxic ischemia.

## Introduction

Perinatal hypoxic ischemic encephalopathy (HIE) is a major cause of neonatal mortality and long-term neurological disorders, including cerebral palsy, mental retardation, epilepsy and learning disabilities (1). In developed and non-developed countries, 2–5 of every 1,000 infants develop perinatal HIE, with 20–40% of these infants suffering from major neurological sequelae and growth retardation (2).

Currently, therapeutic hypothermia is commonly administered to infants with moderate to severe asphyxia (3). While these practices yield promising results, abnormal outcomes have been observed in almost half of the infants treated with therapeutic hypothermia and infants with severe damage have not been saved (4). Previous experimental data have demonstrated that hypothermia extends the duration of the therapeutic window (5,6) and neuroprotection may be reinforced with certain drugs administered during this period (6–8). The therapeutic window following HIE (i.e., the interval during which intervention may be efficacious in preventing ongoing brain injury and/or reducing the severity of ultimate brain injury) is short and considered to be <6 hours (4–6). Currently, research is focused on pre-clinical studies of agents that may exert synergistic activity with hypothermia, with the aim that sequelae-free survival may be improved with a combination (8,9).

Several agents have been shown to be neuroprotective in experimental neonatal HI models, although extremely few agents have been used successfully in clinical practice (9). Cytokine-associated brain injury in HI brain damage is gaining increasing attention. Several cells of the brain (microglia, astrocytes, endothelial cells and neurons) are known to secrete cytokines. Environmental mononuclear phagocytes, T lymphocytes, natural killer cells and polymorphonuclear cells with cytokine synthesizing and releasing capabilities are considered to be capable of crossing the blood-brain barrier, contributing to inflammation and gliosis in the brain (10).

Inflammatory mediators (cytokines and chemokines) have been implicated in the pathogenesis of HIE and may represent a final common pathway of brain injury (11). Animal studies indicate that cytokines, particularly interleukin (IL)-1β, contribute to HI damage. The exact mechanisms by which inflammatory mediators contribute to this process remain unclear (10). Of the proinflammatory cytokines, tumor necrosis factor-α (TNF-α), IL-6 and IL-1β have extremely significant roles in the cytokine cascade. Primary effects of cytokines include endothelial cell activation, leukocyte endothelial adhesion, chemotaxis of leukocytes to inflammation sites, secretion of free oxygen radicals, nitric oxide (NO) synthesis, degranulation, intracellular access of sodium, phagocytosis and procoagulant activity (12).

Although the mechanism of action of immunoglobulin (Ig) is not yet completely understood, Ig is known to be involved in the regulation of Fc receptor expression and function, complement activation and cytokine network activity. In addition, Ig is involved in the provision of anti-idiotypic antibodies, as well as the activation, differentiation and functional regulation of T and B cells (13). Administration of Ig to mice in an induced stroke model has been shown to reduce the infarct volume by decreasing C3b expression at the ischemic site and apoptosis (14). In addition, Ig is involved in reducing post-stroke complement-mediated cell damage and suppressing the activation of microglia and astrocytes, which reduces the release of proinflammatory cytokines and suppresses caspase-3 activation. This in turn reduces the activation of endothelial cells and thus protects the integrity of the blood-brain barrier (15).

The objective of the present study was to evaluate the neuroprotective effects of Ig, an antibody with activity at various stages of the HIE process, which is used in clinical practice for the treatment of several disorders of neonates, in a neonatal HI rat model.

## Materials and methods

### Animals

Seven-day-old Wistar rats (n=40) of either gender, which were delivered spontaneously, were used in this experimental study. Rat pups were obtained from Experimental Animal Unit of Akdeniz University (Antalya, Turkey). The study was performed with the approval of the Ethics Committee of Akdeniz University (Faculty of Medicine, Antalya, Turkey). Data for the control and hypoxia groups were obtained from our recent study (16).

### Animal preparation and surgical procedure

Rat pups were anaesthetized by ether inhalation and the duration of anaesthesia was <5 min. HI brain injury was induced according to the Levine-Rice model (17). Briefly, a median incision was made in the neck and, under microscopic magnification, the left common carotid artery was dissected and ligated with a 6–0 silk suture. Following the suturing of the wound, the animals were allowed a 3-h recovery and feeding period. In control group animals, neither ligation, nor hypoxia was performed (sham surgery). With the exception of the control group, rats were then placed in a plastic chamber and exposed to a continuous flow of 8% oxygen and 92% nitrogen for 2 h. Following the hypoxic period, the rats had a 2-h recovery period in an open chamber without any supplemental oxygen. The animals in the control group were placed in an open chamber for the same intervals. The chambers were partially submerged in a water bath at 37ºC to maintain a constant thermal environment. Following these procedures, all pups were sacrificed by decapitation.

Seven-day-old pups were randomly divided into three groups: Control (n=8), following the median neck incision, neither ligation nor hypoxia was performed; hypoxia (n=16), 0.5 ml saline was injected intraperitoneally immediately following hypoxia; and Ig + hypoxia (n=16; Octagam^®^; Octapharma, Vienna, Austria), rat pups were intraperitoneally administered 1 g/kg Ig immediately following hypoxia. Eight rats from each of the hypoxia and Ig + hypoxia groups were sacrificed 4 and 24 h following drug administration. The rats in the control group were decapitated at the 4 h time-point. Caspase-3 activity and IL-1β, IL-6 and TNF-α mRNA expression levels were studied in the left half of the brain.

### Determination of TNF-α, IL-6 and IL-1β mRNA expression levels by qPCR

Total RNA was isolated from the tissues of each group using a Purelink RNA mini kit (Invitrogen Life Technologies, Carlsbad, CA, USA) according to the manufacturer's instructions. High capacity RNA to cDNA master mix (Applied Biosystems, Foster City, CA, USA) was used for cDNA synthesis in each sample. TaqMan gene expression master mix (Applied Biosystems), TNF-α, IL-6 and IL-1β primers and probes (Taqman Gene Expression Assay numbers: Rn00562055_m1, Rn01410330_m1 and Rn00580432_m1; Applied Biosystems) were used for PCR amplification using the Light Cycler 480 II Real-Time PCR System (Roche Applied Science, Mannheim, Germany). TaqMan PCR was carried out according to the manufacturer's instructions. GAPDH (Taqman Gene Expression Assay number: Rn01775763_g1; Applied Biosystems) was used as a housekeeping gene for this experiment. In order to identify the relative TNF-α, IL-6 and IL-1β mRNA expression levels in the hypoxia and Ig + hypoxia groups, 2^−ΔΔCt^ (fold change) values were calculated, as described previously (18).

### Caspase-3 colorimetric assay

Active caspase-3 levels were measured using a commercial kit (Caspase-3/CPP32 Colorimetric Assay kit; BioVision, Milpitas, CA, USA) according to the manufacturer's instructions. The assay was based on spectrophotometric detection of the chromophore *p*-nitroaniline (pNA) following cleavage from the labeled substrate, Asp-Glu-Val-Asp-pNA. The pNA light emission was quantified using a microtiter plate reader (Multiskan Spectrum, Thermo Labsystems, Franklin, MA, USA) at 405 nm. Values were expressed as arbitrary unit/μg protein.

### Statistical analysis

Data were analyzed using the statistical package program SPSS for Windows version 18.0 (SPSS, Inc., Chicago, IL, USA). Descriptive statistics, including frequency distribution, mean and SD, were used to describe the sample. Kruskal-Wallis variance analysis was utilized to determine inter-group differences; paired groups were compared using a Mann-Whitney U test for analyses yielding significant results; time-dependent variables were analyzed by Wilcoxon test and the correlations among the variables were examined using Spearman's correlation. P<0.05 was considered to indicate a statistically significant difference.

## Results

### Effect of Ig on TNF-α mRNA expression levels in ischemic left hemispheres

**Figure 1:**
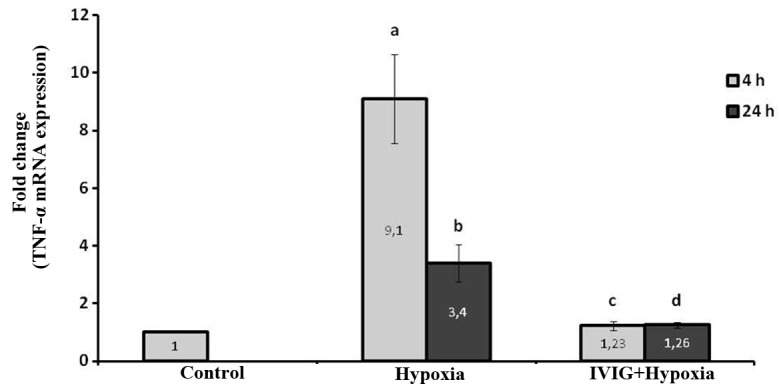
Effect of Ig on TNF-α mRNA expression levels in ischemic left hemispheres of the control, hypoxia and Ig + hypoxia treated rats. ^a^P=0.002, vs. control; ^b^P=0.002, vs. control; ^c^P=0.004, vs. hypoxia; ^d^P=0.004, vs. hypoxia. Ig, immunoglobulin; TNF-α, tumor necrosis factor-α.

Induction of cerebral ischemia increased TNF-α mRNA expression levels significantly at 4 and 24 h, following ischemia in the left ischemic hemispheres in the hypoxia group compared with those in the control (P=0.002 for 4 and 24 h; Fig. 1).

The systemic administration of Ig significantly reduced the TNF-α mRNA expression levels in ischemic tissue at 4 and 24 h following HIE compared with those in the hypoxia group (P=0.004 for 4 and 24 h; Fig. 1). These results indicate that Ig reduced TNF-α production in cortical ischemic tissue following hypoxia.

### Effect of Ig on IL-6 mRNA expression levels in ischemic left hemispheres

**Figure 2:**
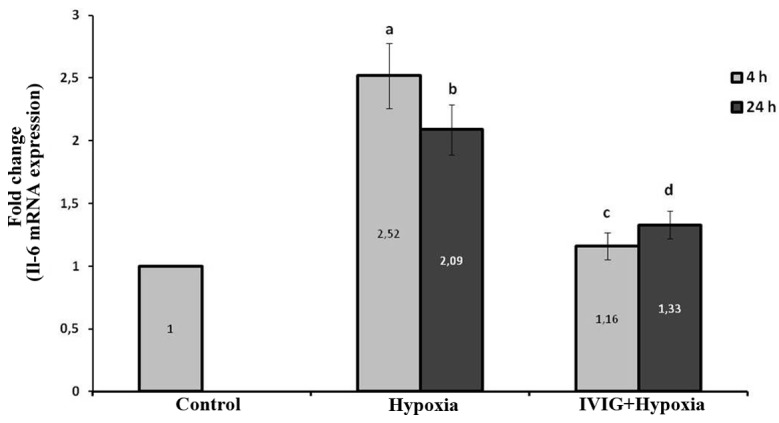
Effect of Ig on IL-6 mRNA expression levels in ischemic left hemispheres of the control, hypoxia and Ig + hypoxia treated rats. ^a^P=0.002, vs. control; ^b^P=0.002, vs. control; ^c^P=0.004, vs. hypoxia; ^d^P=0.004, vs. hypoxia. Ig, immunoglobulin; IL-6, interleukin-6.

The induction of cerebral ischemia increased IL-6 mRNA expression levels significantly at 4 and 24 h following ischemia in the left ischemic hemispheres in the hypoxia group compared with those in the control group (P=0.002 for 4 and 24 h; Fig. 2).

Systemic administration of Ig significantly reduced the IL-6 mRNA expression levels in ischemic tissue at 4 and 24 h following HIE compared with those in the hypoxia group (P=0.004 for 4 and 24 h; Fig. 2). These results indicate that Ig reduced IL-6 production in cortical ischemic tissue following hypoxia.

### Effect of Ig on IL-1β mRNA expression levels in ischemic left hemispheres

The induction of cerebral ischemia increased IL-1β mRNA expression levels significantly at 4 and 24 h following ischemia in the left ischemic hemispheres in the hypoxia group compared with those in the control (P=0.002 for 4 and 24 h; Fig. 2).

**Figure 3:**
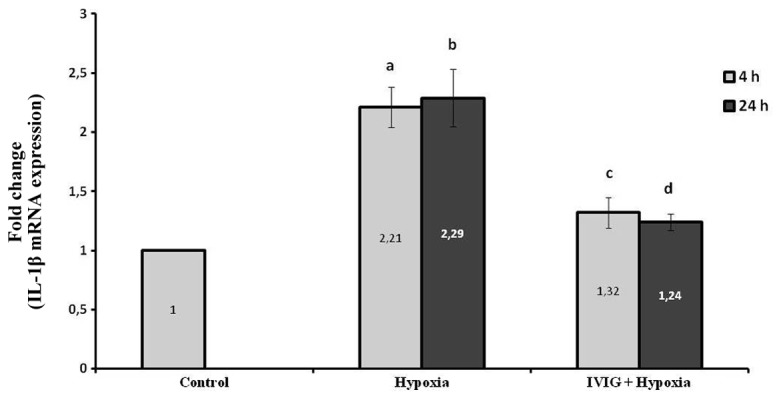
Effect of Ig on IL-1β mRNA expression levels in ischemic left hemispheres of the control, hypoxia and Ig + hypoxia treated rats. ^a^P=0.002, vs. control; ^b^P=0.002, vs. control; ^c^P=0.004, vs. hypoxia; ^d^P=0.004, vs. hypoxia. Ig, immunoglobulin, IL-1β, interleukin-1β.

Systemic administration of Ig significantly reduced the IL-1β mRNA expression levels in ischemic tissue at 4 and 24 h following HIE compared with those in the hypoxia group (P=0.01 for 4 h and P=0.005 for 24 h; Fig. 3). These results indicate that Ig reduced IL-1β production in cortical ischemic tissue following hypoxia.

### Effect of Ig on caspase-3 activities in ischemic left hemispheres

**Figure 4:**
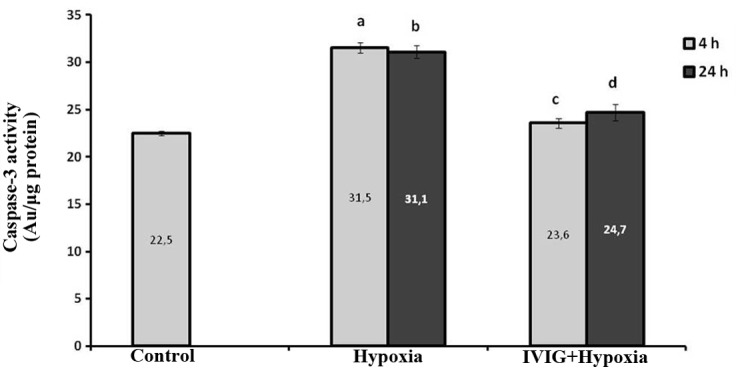
Effect of Ig on caspase-3 activity in ischemic left hemispheres of the control, hypoxia and Ig + hypoxia treated rats. ^a^P=0.004 vs. control; ^b^P=0.004, vs. control; ^c^P=0.004, vs. hypoxia; ^d^P=0.004, vs. hypoxia. Ig, immunoglobulin.

As shown in Fig. 4, caspase-3 activity in the left half of the brains in the hypoxia group were found to have increased significantly compared with those in the control group (P=0.004 for 4 and 24 h). Caspase-3 activities in the brains of the Ig + hypoxia group were significantly lower than those of the hypoxia group (P=0.004 for 4 and 24 h; Fig. 4).

## Discussion

In previous studies, the latent phase between the HI event and cell death was found to be extremely critical in HI injury and this period was named the ‘therapeutic window’ (4–8). This period may range between 6 and 12 h in a neonate who has suffered from hypoxia and ischemia. Terminating the cascade of molecular events that develop during the therapeutic window phase and lead to cell death or limiting the cascade at a certain point is extremely critical in reducing or preventing the sequelae (9).

It is well established that excitotoxicity and free radical production have negative contributions to the early ischemic response. However, significant functional improvements have not been achieved in clinical and experimental studies where various agents were administered selectively for treatment or stopping progression during the stages of this mechanism. A second wave of necrosis occurs in response to an ischemic attack, which is mediated by the neuroinflammatory response to the damage (19). Accumulated evidence demonstrates that targeting the late neuroinflammatory response with the treatment may be a promising approach for therapeutic intervention (19,20).

Rat models of HI injury provide a convenient method for understanding histopathological and biochemical outcomes, as well as the long-term neurological impacts of HIE (21). Rats aged 7 days were used in the present study. The brains of a 7-day-old rat litter were considered to be relevant for the perinatal period in humans, particularly with regard to brain development in the latter species (22).

Previous studies have shown increased numbers of apoptotic neurons in both brain hemispheres, with a more pronounced increase in the hemisphere with carotid artery blockage, following ischemia and 1–3 h hypoxia in neonatal rats. These studies indicated that the acute effects of HIE may be managed with agents that reduce apoptosis (23,24). In the present study, a significant increase was observed in caspase-3 levels in the hypoxia group compared with those in the control at 4 and 24 h following HI induction.

The pathophysiological role of inflammatory cytokines in the development of HI brain damage has been studied in clinical and experimental studies. Overall, IL-1β has been shown to potentiate ischemic brain damage. Rapid elevations of IL-1β levels in the brain tissue have been demonstrated following HI injury or transient mid-cerebral artery occlusion in neonatal rats (25,26). In the present study, increased IL-1β mRNA expression was observed following 4 and 24 h in the hypoxia group compared with the control.

In addition, TNF-α, IL-6 and IL-1β are known to be the major inflammatory mediators with increased levels in HIE. These cytokines are released by peripheral monocytes and macrophages, as well as by astrocytes and microglia of the central nervous system (26,27). The primary effects of cytokines include endothelial cell activation, leukocyte endothelial adhesion, chemotaxis of leukocytes to an inflammation site, secretion of free oxygen radicals, NO synthesis, degranulation, intracellular sodium access, phagocytosis and procoagulant activity (28).

TNF-α is known to be an apoptosis activator that potentiates Fas expression and potentially results in neuronal apoptosis (22). An experimental model has also shown that there is a transient increase in IL-1β and TNF-α levels, peaking 6 h following hypoxia and that brain damage was prevented with TNF-α inhibition (29). Similarly, in the present study, increased TNF-α mRNA expression levels were observed at 4 and 24 h in the hypoxia group compared with those in the control group and apoptosis was reduced by the suppression of elevations in TNF-α mRNA expression in the treatment groups.

Given the aforementioned benefits of Ig treatment, studies have investigated its effects on the inflammatory cascade and apoptosis by inducing stroke in adult rats. Arugman *et al* (14) reported almost complete elimination of mortality and a 50–60% reduction in infarct size with Ig administration in adult rats exposed to experimental stroke.

To the best of our knowledge, there are no experimental studies in the literature examining the effects of Ig on neonatal HIE and there are only two clinical studies. Chen *et al* (30) compared the efficacy of Ig with that of routine treatment in neonates with HIE. The authors reported improvements in abnormal primitive reflex duration and muscle tone, the elimination of convulsions and a shorter duration of hospitalized care for the group treated with Ig compared with the group receiving routine treatment. The authors also concluded that Ig alleviated brain damage and multi-organ dysfunction and that HIE duration was shortened by the inhibition of IL-6 and TNF-α production. In a similar study by Dong *et al* (31), levels of IL-6, 8 and 10 decreased significantly on day 3 relative to those on day 0 in neonates with HIE treated with Ig. Decreased levels were not observed in the hypoxic group without Ig treatment. The authors therefore hypothesized that Ig treatment may provide a short-term improvement of brain damage in neonates with HIE.

In the present experimental model, Ig was selected as an anti-inflammatory agent to prevent cerebral apoptosis by reducing or preventing an inflammatory response. The effects of Ig on cerebral apoptosis in a neonatal HI rat model were evaluating in this novel study. The observations indicate that Ig administration may be an efficient treatment approach for reducing cerebral apoptosis, based on significantly lower IL-6, IL-1β and TNF-α mRNA expression levels and caspase-3 activity in the animals treated with Ig, as measured at 4 and 24 h following HI injury with a colorimetric method. Ig contains high-affinity neutralizing antibodies against IL-1β, IL-6 and TNF-α in quantities that are sufficient to suppress circulating proinflammatory pathogenic cytokines or downregulate the synthesis of cytokines by T cells (32). The modulation of cytokines and cytokine antagonists by Ig is another major mechanism by which Ig exerts its anti-inflammatory effects. Ig has been shown to selectively trigger the production of IL-1 receptor antagonist, the natural antagonist of IL-1 (33).

In the present study, a correlation analysis between TNF-α/IL-1β mRNA expression levels and infarct size was not performed as the volumes of the infarct sizes were not measured. The aim was to ascertain cytokine gene expression and caspase-3 activation in the area of infarction; therefore no examinations were performed on the contralateral hemisphere.

In conclusion, the experimental model of the present study indicated that Ig therapy reduced caspase-3 activity, thereby reducing apoptosis to a significant extent. Ig also reduced TNF-α, IL-6 and IL-1β expression. Ig may also provide therapeutic effects in stroke through the inhibition of cytokines and the subsequent infiltration of inflammatory cells, thus reducing inflammation in the region of infarction. Traditional treatment of HIE is supportive care. Therapeutic hypothermia has become common practice in a number of institutions since a benefit in moderate to severe encephalopathic newborns has been observed; however, it does not completely protect or repair an injured brain; therefore, the search for adjuvant therapies continues. Ig may be a candidate drug for combining with therapeutic hypothermia in the treatment of HIE. However, further studies are required to investigate this.
